# Gradients of striatal function in antipsychotic-free first-episode psychosis and schizotypy

**DOI:** 10.1038/s41398-023-02417-2

**Published:** 2023-04-18

**Authors:** Marianne Oldehinkel, Jeggan Tiego, Kristina Sabaroedin, Sidhant Chopra, Shona M. Francey, Brian O’Donoghue, Vanessa Cropley, Barnaby Nelson, Jessica Graham, Lara Baldwin, Hok Pan Yuen, Kelly Allott, Mario Alvarez-Jimenez, Susy Harrigan, Christos Pantelis, Stephen J. Wood, Patrick McGorry, Mark A. Bellgrove, Alex Fornito

**Affiliations:** 1 Turner Institute for Brain and Mental Health, School of Psychological Sciences, and Monash Biomedical Imaging, Monash University, Clayton, Australia; 2 Donders Institute for Brain, Cognition and Behaviour, Radboud University Medical Center, Nijmegen, Netherlands; 3 Orygen Youth Health, Parkville, Australia; 4 Centre for Youth Mental Health, University of Melbourne, Melbourne, Australia; 5 Department of Social Work, Monash University, Melbourne, Australia; 6 Melbourne School of Population and Global Health, University of Melbourne, Melbourne, Australia; 7 Melbourne Neuropsychiatry Centre, Department of Psychiatry, The University of Melbourne, Melbourne, Australia; 8 School of Psychology, University of Birmingham, Birmingham, UK

**Keywords:** Schizophrenia, Diagnostic markers

## Abstract

Both psychotic illness and subclinical psychosis-like experiences (PLEs) have been associated with cortico-striatal dysfunction. This work has largely relied on a discrete parcellation of the striatum into distinct functional areas, but recent evidence suggests that the striatum comprises multiple overlapping and smoothly varying gradients (i.e., modes) of functional organization. Here, we investigated two of these functional connectivity modes, previously associated with variations in the topographic patterning of cortico-striatal connectivity (first-order gradient), and dopaminergic innervation of the striatum (second-order gradient), and assessed continuities in striatal function from subclinical to clinical domains. We applied connectopic mapping to resting-state fMRI data to obtain the first-order and second-order striatal connectivity modes in two distinct samples: (1) 56 antipsychotic-free patients (26 females) with first-episode psychosis (FEP) and 27 healthy controls (17 females); and (2) a community-based cohort of 377 healthy individuals (213 females) comprehensively assessed for subclinical PLEs and schizotypy. The first-order “cortico-striatal” and second-order “dopaminergic” connectivity gradients were significantly different in FEP patients compared to controls bilaterally. In the independent sample of healthy individuals, variations in the left first-order “cortico-striatal” connectivity gradient were associated with inter-individual differences in a factor capturing general schizotypy and PLE severity. The presumed cortico-striatal connectivity gradient was implicated in both subclinical and clinical cohorts, suggesting that variations in its organization may represent a neurobiological trait marker across the psychosis continuum. Disruption of the presumed dopaminergic gradient was only noticeable in patients, suggesting that neurotransmitter dysfunction may be more apparent to clinical illness.

## Introduction

Altered striatal functioning, in particular increased dopaminergic signaling in the striatum, has long been implicated in the pathophysiology of psychotic disorders [1, 2]. The ventral striatum (ventral caudate and nucleus accumbens) and dorsal striatum (dorsal caudate and putamen) receive dense dopaminergic projections from the ventral tegmental area (VTA) and substantia nigra (SN), respectively [3]. Positron emission tomography (PET) studies in patients with psychotic disorders have consistently demonstrated increased dopaminergic signaling in the striatum, as indexed by elevated presynaptic dopamine synthesis capacity [4], exaggerated dopamine release following amphetamine challenge [5], and increased postsynaptic dopamine D_2_ receptors [6].

These findings complement resting-state functional magnetic resonance imaging (fMRI) studies implicating altered function of cortico-striatal networks in psychotic disorders, particularly in the dorsal (or associative/cognitive) system linking the dorsolateral prefrontal cortex (DLPFC) and thalamus to the dorsal striatum [7, 8]. Decreased functional connectivity between caudate, thalamus and/or DLPFC has repeatedly been reported in patients with schizophrenia [9–11], and in patients with first-episode psychosis [12] compared to controls. Some studies have also reported alterations in the ventral (or limbic) cortico-striatal system that connects the orbitofrontal cortex (OFC), medial prefrontal cortex, and limbic structures with the ventral striatum [8, 13] in patients with psychosis [10, 12, 14], but the findings are more heterogeneous. For example, Fornito et al. [12] and Sarpal et al. [14] reported increased connectivity between ventral striatum and OFC and insula in patients, while Peters et al. [10] reported decreased connectivity of ventral striatum with left anterior insula.

There is also emerging evidence suggesting that cortico-striatal systems may be related to the expression of subclinical psychosis-like experiences (PLEs), such as distortions of thinking and perception, cognitive impairments, flattened affect, and difficulties in social functioning. These experiences, which are defining features of schizotypy, are thought to vary along a spectrum of severity in the general population and are believed to represent a continuum of risk for (clinically-significant) psychotic illness [15–18]. Mirroring findings in clinical populations, people with higher levels of PLEs, and individuals experiencing an at-risk mental state (ARMS), show lower functional connectivity within the dorsal cortico-striatal circuit [19–21]. PLEs have also been linked to increased connectivity within the ventral circuit [21]. Interestingly, a PET study in healthy individuals with frequent persistent auditory hallucinations did not find evidence of increased striatal dopamine synthesis capacity [22], suggesting that while cortico-striatal functional connectivity may relate to both the subclinical and clinical expression of psychosis, dopaminergic dysfunction may only manifest in clinically significant illness.

One limitation of these studies is that they rely on a discrete parcellation of striatum into distinct subregions. Instead, recently developed gradient-based approaches can be used to characterize continuous variations of neural organization across diverse cortical [23–25] and subcortical areas [26–29]. In particular, functional connectivity gradients can be obtained with high reproducibility and reliability, and can predict phenotypic variations with higher accuracy than connectivity measures derived from traditional parcellation-based approaches, making them of interest for potential biomarker development [30]. Indeed, in recent work applying connectopic mapping [23], a novel gradient-based method for characterizing continuous variations of functional organization to resting-state fMRI data, we showed that striatal function is organized along distinct yet spatially overlapping continuous gradients that tap into different aspects of striatal function [26, 29] that are missed by discrete parcellation-based analyses. Notably, three key gradients of the striatum have been defined: a dominant (zero-order) mode of functional connectivity which represents the anatomical subdivision of striatum in putamen, caudate and nucleus accumbens (NAcc); a first-order mode constituting a ventromedial-to-dorsolateral gradient associated with cortico-striatal-mediated goal-directed behavior, ranging from emotional and reward-related functions ventromedially to associative and motor functions dorsolaterally; and a second-order mode that shows a high spatial correspondence with dopaminergic projections to striatum (see supplementary Figure S1; [26, 29]), suggesting that this gradient can provide a non-invasive, functional MRI-based probe of striatal dopamine function.

Here, we used connectopic mapping to identify smoothly varying and spatially overlapping topographic connection patterns (‘connectivity modes’) in the striatum in a sample of clinically diagnosed, antipsychotic-free first-episode psychosis (FEP) patients and an independent sample of non-clinical individuals from the community who were comprehensively assessed for diverse aspects of schizotypy and PLEs. Our approach thus allowed us to examine continuities in the functional organization of striatum between frank psychotic disorder and subclinical traits and experiences associated with illness risk. Given evidence for the involvement of cortico-striatal connectivity across the continuum [19, 20], but dopaminergic dysfunction specifically in clinical illness [22], we hypothesized that the first-order cortico-striatal mode would be implicated in both clinical and subclinical groups whereas the second-order dopaminergic mode would only be implicated in the clinical cohort.

## Materials and methods

### FEP dataset

#### Study design and participants

A total of 61 (28 females) antipsychotic-free FEP patients aged 15–25 years, and 27 (17 females) healthy controls aged 18–25 years participated in the study [31], which was part of a clinical trial (ACTRN12607000608460), yet only data at baseline was selected for this study. All patients were experiencing a first episode of psychosis meeting the Structured Clinical Interview for DSM-5 (SCID; [32]) criteria for a psychotic disorder, including schizophrenia, schizophreniform disorder, delusional disorder, brief psychotic disorder, major depressive disorder with psychotic symptoms, substance-induced psychotic disorder or psychosis not otherwise specified. Participants were excluded for contraindications to MRI, a duration of untreated psychosis >6 months, significant prior exposure to antipsychotic medication (>7 days of use or >1750 mg lifetime chlorpromazine equivalent exposure; with none taking antipsychotic medication at the time of scanning), not being able to provide informed consent, and insufficient comprehension of the English language. Healthy controls had never used psychotropic medication and had no personal or first-degree family history of a psychiatric or neurological condition. Informed consent was obtained from all participants and the study was approved by local ethics committees. Further details about recruitment procedures are provided elsewhere [33]. Demographic and clinical details are provided in Table 1.

**Table 1:** Sample characteristics of the FEP dataset.

	Psychosis patients (*N* = 56)		Healthy controls (*N* = 27)		T / χ^2 (a)^	*p* ^(b)^
Baseline age, years *(SD)*	19.1	*2.87*	21.9	*1.93*	−4.43	*<0.001*
Females, N *(%)*	26	*46.6%*	17	*62.9%*	2.00	*0.170*
Handedness, left, N *(%)*	3	*5.36%*	3	*11.1%*	0.37	*0.677*
Education, years *(SD)*	12.2	*2.13*	15.2	*1.9*	−6.15	*<0.001*
Head motion, meanFD *(SD)*	0.052	*0.041*	0.074	*0.053*	−2.04	*0.045*
Diagnosis, N						
Major depression with psychosis	11					
Schizophreniform disorder	9					
Psychotic disorder NOS	15					
Substance-induced psychotic disorder	5					
Delusional disorder	5					
Schizophrenia	10					
Missing diagnosis	1					
BPRS total score, mean *(SD)*	57.3	*10.0*				
BPRS positive subscale score, mean *(SD)*	16.1	*4.3*				
SANS total score, mean *(SD)*	34.6	*17.2*				

Abbreviations: *meanFD* mean framewise displacement, *NOS* not otherwise specified, *BPRS* Brief Psychiatric Rating Scale version 4, *SANS* Scale for Assessment of Negative Symptoms.

^a^This column provides the T or *χ*^2^ values comparing the healthy control group and patient group at baseline.

^b^The corresponding *p*-value is shown in the last column.

#### Clinical assessment

Patients were rated by a clinician on the Brief Psychiatric Rating Scale version 4 (BPRS-4; [34]) and the Scale for Assessment of Negative Symptoms (SANS; [35]). We selected total scores on the BPRS-4, scores on the positive symptom subscale from the BPRS-4, and the total SANS scores for further analyses of total, positive, and negative symptom severity, respectively. These scores were selected as clinical analogues to the PLEs score estimates obtained in the community sample, see below.

#### MRI data acquisition and preprocessing

MRI data were acquired on a 3 T Siemens Trio Tim scanner located at the Royal Children’s Hospital in Melbourne, Australia. Structural images were obtained using a 6-min T1-weighted Magnetisation-Prepared Rapid Gradient Echo (MPRAGE) sequence (TR = 2300 ms, TE = 2.98 ms, voxels size = 1.1 × 1.1 × 1.2 mm, flip angle = 9**°**, matrix size = 256 × 256, FOV = 256 mm, 176 sagittal slices). An 8-min resting-state fMRI scan was acquired using an interleaved echo planar imaging (EPI) sequence (TR = 2000 ms, TE = 32 ms, flip angle = 90**°**, matrix size = 64 × 64, voxel size = 3.3 × 3.3 × 3.55 mm, FOV = 210 mm, 37 axial slices, slice thickness = 3.5 mm, volumes = 234). Participants were instructed to lie still in the scanner while maintaining wakefulness with eyes closed for the duration of the resting-state fMRI scan. The resting-state fMRI data were preprocessed using fMRIPrep [36]. Preprocessing included removal of the first five volumes to allow for signal equilibration, primary head motion correction via realignment to the middle volume (MCFLIRT; [37]), slice-time correction, grand mean scaling, and spatial smoothing with a 4 mm FWHM Gaussian kernel. Following distortion correction using fieldmaps, EPIs were co-registered with their corresponding T1w using boundary-based registration with nine degrees of freedom using the bbregister routine in FreeSurfer. The motion-correcting transformations, field-distortion-correcting warp, EPI-to-T1w transformation, and T1w-to-MNI template warp were concatenated and applied in a single step using ANTs version 2.1.0. All subsequent preprocessing steps and analyses were conducted in MNI152 standard space. Next, ICA-AROMA, an ICA-based method that automatically detects and removes motion-related components from the data [38] was applied to thoroughly correct for secondary head-motion related artifacts [39]. We then applied nuisance regression to remove signal from white matter and cerebrospinal fluid, and a high-pass filter (0.01 Hz). Participants with poor imaging data (*N* = 4) and/or excessive head motion during the resting-state fMRI scan were excluded (*N* = 1), as per the following criteria: mean root mean squared of the frame wise displacement (meanFD) > 0.2, sum of suprathreshold spikes > 20%, or any FD > 5 mm [39]. The final sample thus comprised 56 patients and 27 controls. The clinical and demographic characteristics of both groups are listed in Table 1.

### Community dataset

#### Study design and participants

719 Right-handed Caucasian adults (424 females) aged 18–50 years were recruited from the general community. Participants had no personal history of psychiatric or neurological illness and no intellectual disability. The Monash University Human Research Ethics Committee approved the study. For more details about this sample, see the first paragraph of the supplementary material.

#### PLE and schizotypy measures

Participants completed an online survey including eleven different measures of PLEs and schizotypal traits, comprising a total of 251 items. These measures consisted of the Peters Delusions Inventory-21 (PDI-21; [40]), the Oxford-Liverpool Inventory of Feelings and Experiences Short Form (sO-LIFE; [41]) consisting of subscales for unusual experiences, cognitive disorganization, introvertive anhedonia, and impulsive nonconformity, the Wisconsin Schizotypy Scales (WSS) comprised of subscales for magical ideation, physical anhedonia, social anhedonia and perceptual aberration [42], and the Community Assessment of Psychic Experience (CAPE; [43]), including subscales for positive, negative and depressive symptoms. We selected a wide range of schizotypy measures to avoid reliance on specific theoretical conceptualization of the construct. The measures we used span both clinical (i.e., WSS & PDI-21) and psychometric/personality conceptualizations (i.e., CAPE & sO-LIFE). The inclusion of items from taxonic (i.e., WSS, PDI-21) and dimensional (i.e., CAPE, sO-LIFE) perspectives also had the practical advantage of increasing the reliability of measurement across the latent trait continuum.

We used a bifactor structural equation model [44] to estimate three latent, orthogonal symptom dimensions summarizing phenotypic variation in schizotypy across the eleven subscales: (1) a *General* schizotypy factor capturing a general tendency to experience positive, negative, and disorganized PLEs and express diverse schizotypal traits; (2) a *Positive* schizotypy factor representing residual shared variance unique to the experience of positive PLEs and schizotypy, such as disturbances in thought and perception; and (3) a *Negative* schizotypy factor capturing residual shared variance unique to the experience of negative PLEs and schizotypy, such as anhedonia and social deficits. Note that while here we refer to these dimensions as general, positive, and negative aspects of schizotypy for convenience, the dimensions capture variance related to both schizotypy and PLEs. Following model estimation, factor score estimates were generated using the regression method of [45] and used for subsequent analysis. See the supplementary material for a detailed description of this procedure.

#### MRI data acquisition and preprocessing

A total of 415 participants also completed a structural and functional MRI scan, acquired on a 3 T Siemens Skyra scanner located at the Monash Biomedical Imaging (MBI) facility in Melbourne, Australia. Structural images were obtained using a 7-min T1-weighted MPRAGE sequence (TR = 2300 ms, TE = 2.07 ms, voxels size = 1 mm^3^, flip angle = 9**°**, matrix size = 256 × 256, FOV = 256 mm, 192 sagittal slices). An 8-min multiband resting-state fMRI was acquired using an EPI sequence equipped with a 32-channel head coil (TR = 754 ms, TE = 21 ms, flip angle = 50**°**, multiband acceleration factor = 3, matrix size = 64 × 64, in-plane resolution = 3 mm^3^, FOV = 190 mm, 42 axial slices, slice thickness = 3 mm, volumes = 620). Participants were instructed to relax and keep their eyes closed for the duration of the resting-state fMRI scan. We selected all participants with complete schizotypy data for whom a structural and resting-state fMRI scan were available (*N* = 407). Participants with excessive head motion during the resting-state fMRI scan, defined using the same criteria applied to the FEP dataset, were also excluded (*N* = 30). The final resting-state fMRI sample thus comprised 377 individuals (213 females) aged between 18 and 50 years (*M* = 23.3, *SD* = 5.18). Preprocessing of the data was identical to the preprocessing procedure of the FEP dataset.

### Connectopic mapping

**Fig. 1:**
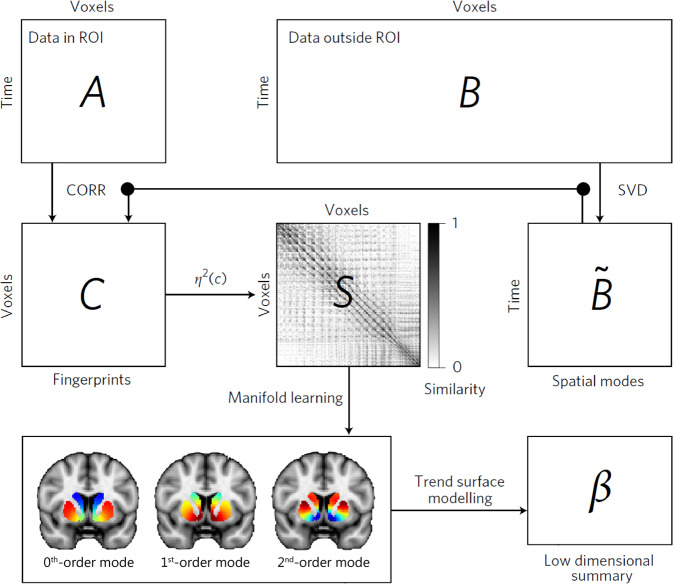
The connectopic mapping pipeline. The fMRI voxelwise time-series from a pre-defined striatal region-of-interest (ROI) are rearranged into a time-by-voxel matrix *A*, as are the time series from all voxels outside the ROI (matrix *B*). The dimensionality of *B* is losslessly reduced using SVD, yielding $\tilde B$. The connectivity fingerprint of every voxel within the ROI is computed as the Pearson’s correlation (CORR) between its time-series and the SVD-transformed data, yielding matrix *C*. The similarity in connectivity profiles between voxels is then quantified using the *η*^2^ coefficient, resulting in matrix *S*. Laplacian eigenmap manifold learning is then used to yield a set of connection topographies or “connectivity modes” that together describe continuous spatial gradients in the functional organization of the striatum. Voxels that have similar colors in these connectivity modes have similar connectivity patterns with the rest of the brain, with red and blue colors indicating voxels that are maximally different from each other in terms of their connectivity pattern. Finally, trend surface modeling is applied to summarize the connectivity gradients by fitting a set of trend coefficients (*β*) that optimally combine a set of spatial polynomial basis functions.

Gradients of striatal function in both the FEP and community dataset were characterized using connectopic mapping, a novel method that enables characterization of the dominant modes of spatial variations in functional connectivity within the striatum (see Fig. 1; [23]). As in previous work [29], we applied connectopic mapping to the left and right putamen and caudate-NAcc regions separately. Masks for the striatal regions were obtained by thresholding the respective regions from the Harvard-Oxford atlas at 25% probability. In brief, we rearranged the fMRI time-series data from each striatal subregion and all grey-matter voxels outside the striatum into two time-by-voxel matrices. This matrix was subjected to a lossless singular value decomposition (SVD) and correlations between the time-series data of each voxel in the striatal ROI and the SVD-transformed data from outside the striatum were estimated. The *η*^2^ coefficient was then used to quantify similarities between these voxel-wise “fingerprints” [23]. Next, we applied the Laplacian eigenmap non-linear manifold learning algorithm [46] to the acquired similarity matrix, which resulted in a series of vectors representing the dominant modes of functional connectivity change for each subject.

We focused our analyses on the first-order and second-order striatal connectivity modes, which are visualized in Figure 1 and Figure S2, as these have particular relevance for psychosis. More specifically, the first-order mode defines a spatially continuous gradient that has been associated with known cortico-striatal circuitry ranging from limbic ventromedial systems (indicated by red voxels) to associative and motor dorsolateral systems (indicated by blue voxels) [26]. The second-order mode captures a spatial gradient that has recently been associated with dopaminergic innervation of the striatum, as revealed by a high spatial correlation (*r* = 0.884) between the gradient and DAT-SPECT-derived dopamine transporter (DAT) availability [29], a marker of dopaminergic projections. Within this connectivity gradient, red voxels mapped onto high DAT availability (i.e., high density of dopaminergic projections) and blue voxels on low DAT availability (i.e., a low density of dopaminergic projections). We excluded 1 ≤ *N* ≤ 5 datasets for the first-order mode and 21 ≤ *N* ≤ 38 datasets for the second-order mode due to low quality (Table S7), with the numbers depending on the cohort (i.e., FEP or community) and hemisphere (i.e., left or right). As in previous work [29], low data quality was defined as a spatial correlation below 0.5 with the group-average connectivity modes obtained in a prior analysis of the high-quality HCP dataset (see Table 1; [29]). The higher number of excluded gradients in the FEP compared to community dataset is inherent to the lower temporal and spatial resolution of the resting-state fMRI data in the FEP dataset. Similarly, the higher number of exclusions for the second-order gradient can be explained by the slightly lower stability of the second-order (compared to first-order) gradient, which is masked by the connectivity of the first-order gradient. For more details, see [26, 29].

To enable statistical analysis over these connection topographies, we combined the connectivity modes obtained for putamen and caudate-NAcc and fitted spatial statistical models in order to obtain a set of coefficients summarizing the first-order and second-order spatial gradients in the X, Y and Z axes of the MNI152 coordinate space. More specifically, we used trend surface modeling (TSM; [47]), which involves fitting a set of polynomial basis functions defined by the coordinates of each striatal location to predict each subject’s connection topography. We fitted these models using Bayesian linear regression [48]. To select the degree of the interpolating polynomial basis set, we fitted these models across polynomials of degrees ranging from 1 to 5 and then compared the different model orders using a Scree plot analysis [49]. This criterion strongly favored a polynomial of degree 3 for both the first and second order connectivity modes, see Supplementary Figures S3 and S4. This means that the connectivity mode in striatum was modelled with linear, quadratic, and cubic functions in the *X*, *Y* and *Z* direction of MNI152 coordinate space (9 TSM coefficients). The TSM coefficients of the fitted polynomial basis functions *together* describe the rate at which the gradient changes along a given spatial dimension and can be used for statistical analysis. We excluded subjects with outlying values (defined as 3 standard deviations beyond the mean) in one or more of the TSM coefficients (0 ≤ *N* ≤ 3 individuals, depending on the specific analysis). The polynomials summarized the connectivity modes well, explaining the following proportions of variance in the FEP dataset (mean ± SD): left striatum first-order mode: 88.65 ± 6.40%, right striatum first-order mode: 87.93 ± 6.75%, left striatum second-order mode: 78.95 ± 7.73%, right striatum second-order mode: 78.51 ± 7.61%. Similar results were obtained for the schizotypy dataset: left striatum first-order mode: 91.17 ± 3.56%, right striatum first-order mode: 91.25 ± 3.80%, left striatum second-order mode: 77.32 ± 5.82%, right striatum second-order mode: 78.52 ± 5.30%. In the supplementary material (pages 5 and 6) we further demonstrate the validity of the TSM models by showing that our TSM models are not overfitting the data and the TSM coefficients are not collinear.

### Statistical analyses

We compared the first-order and second-order striatal connectivity modes between the first-episode psychosis group and control group of the FEP dataset by conducting omnibus tests of the TSM coefficients modeling the connectivity modes. More specifically, group differences in the TSM coefficients were assessed using a likelihood ratio test in the context of a logistic regression. Age and gender were included as nuisance covariates. We corrected for multiple comparisons using a Bonferroni-corrected *α* = 0.0125 (corrected for 2 connectivity modes each in the left and right hemisphere) and report the *χ*^2^ (likelihood test) with corresponding *p*-value and the group classification accuracy of tests that revealed significant group differences. For the connectivity modes demonstrating significant group differences, we investigated associations with symptom severity in the FEP group via separate multiple linear regression models that included the TSM coefficients modeling the connectivity modes to predict positive symptom scores (BPRS-positive subscale), negative symptom scores (total SANS score), and total symptom severity scores (total BPRS score), with age and gender as nuisance covariates. As we found that all modes differentiated the groups, these analyses used a Bonferroni *α* = 0.0167 (corrected for the three investigated symptom domains).

Next, we investigated associations between the first-order and second-order connectivity modes with general, positive, and negative schizotypy factor score estimates in the community dataset. We fitted separate multiple linear regression models for each of the three schizotypy dimensions, with the TSM coefficients of the connectivity modes as predictors and age and gender as nuisance covariates. This analysis was again conducted separately for the first-order and second-order connectivity mode and separately for the left and right hemisphere, using a Bonferroni corrected *α* = 0.004 (corrected for 2 connectivity modes in 2 hemispheres across 3 schizotypy dimensions, yielding 12 comparisons in total). For connectivity modes showing significant associations with symptom severity in the FEP cohort and/or schizotypy in the community dataset, we conducted post-hoc partial correlation analyses. In these post-hoc analyses, we controlled for age, gender and the remaining TSM coefficients to assess which TSM coefficients most strongly contributed to the observed associations.

### Analysis of motion and age contamination

In addition to our stringent quality control procedures (see above), we conducted additional post-hoc analyses to test for any residual effects of head motion and effects of age on the findings. For all significant group differences and associations observed in this study, we conducted post-hoc analyses to demonstrate that these findings were not driven by age or head motion. First, meanFD was added as a nuisance covariate to the binary logistic regression or GLM (age was already included in the main analysis), and second, correlations were computed between the TSM coefficients and age, meanFD, and DVARS.

## Results

### Cortico-striatal and dopaminergic gradients are disrupted in FEP patients

**Fig. 2:**
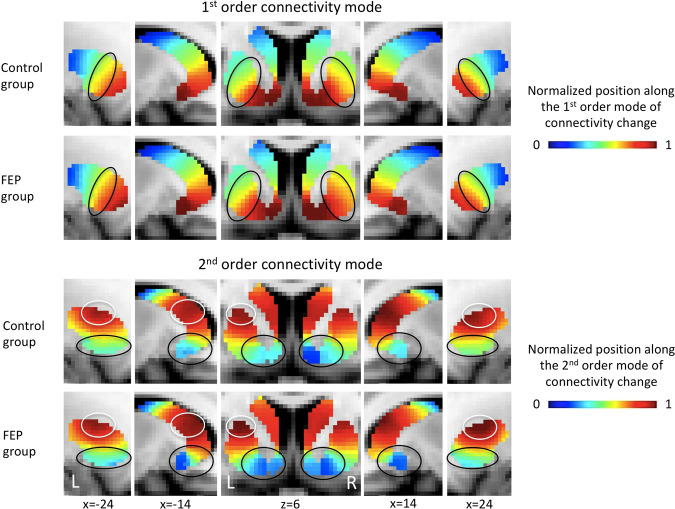
Disrupted striatal functional connectivity gradients in FEP. Images represent the group-average connectivity modes. Voxels that have similar colors in these connectivity modes have similar connectivity patterns with the rest of the brain, with red and blue colors indicating voxels that are maximally different from each other in terms of their connectivity pattern. Please note that omnibus tests of the TSM coefficients summarizing these connectivity modes revealed that the FEP and control group were significantly different, and that these figures have purely been added for visualization purposes. For the first-order connectivity mode, we can see that group differences are mainly evident in the mid-section of the gradient in the putamen region of striatum (circled). For the second-order connectivity mode, differences are evident in both the dorsal (circled in white) and ventral regions (circled in black) of striatum. L left, R right.

Our results show that the first-order mode (left striatum: *χ*^2^ = 33.84, *p* < 0.001, classification accuracy (acc) = 80.5%; right striatum: *χ*^2^ = 29.25, *p* = 0.002, acc = 74.7%) and second-order mode (left striatum: *χ*^2^ = 29.95, *p* = 0.002, acc = 80.6%; right striatum: *χ*^2^ = 27.72, *p* = 0.004, acc = 87.1%) significantly differentiate patients with psychosis compared to controls in both hemispheres after correction for multiple comparisons. Figure 2 shows that these statistical group differences can be observed in the mid-section of the first-order gradient in the putamen region (circled), where patients show a more gradual (i.e., a slower) change in organization from ventral to dorsal striatum. Since the first-order gradient maps onto cortico-striatal connectivity, these alterations might suggest that, in FEP patients, a larger area of the striatum is dedicated to cortico-striatal connectivity typically associated with the more ventral parts of the striatum (the ventral circuit, represented by red-yellow voxels), and a smaller area to connectivity typically associated with more dorsal parts of striatum (the dorsal circuit, represented by blue-green voxels).

Figure 2 also indicates that, for the second-order mode, patients showed a sharper gradient in most areas of the ventral striatum (black circles; more dark blue voxels). In addition, patients showed stronger segregation of the dorsal striatum (white circles; more dark red voxels). This second-order mode maps onto a gradient of dopaminergic innervation from ventral to dorsal striatum [29]. The pattern identified in patients is consistent with more “extreme” values at both the dorsal and ventral ends of the connectivity mode. We speculate that this finding might be consistent with reduced dopamine-mediated functional connectivity of the ventral striatum relative to the dorsal striatum in FEP patients compared to controls [29].

### Gradients of striatal function correlate with symptom severity in FEP

**Fig. 3:**
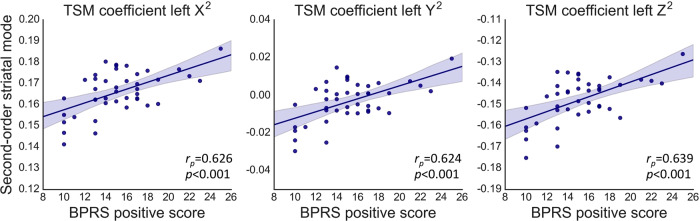
Associations between BPRS positive subscale scores and the second-order connectivity mode in left striatum in patients with first-episode psychosis. To visualize these associations, partial correlations (*r*_p_) are shown between BPRS positive scores and the TSM coefficients that most strongly contributed. The shaded areas represent the confidence intervals of these correlations.

In the patient group, the second-order connectivity mode in left striatum was associated with the BPRS positive symptom scale after correction for multiple comparisons (*F*(11,54) = 2.671, *p* = 0.016, *R*^*2*^ = 0.495). Post-hoc partial correlation analyses revealed that this effect was driven by the quadratic TSM coefficients modeling the connectivity mode in the X (*r*_*p*_ = 0.626, *p* < 0.001), Y (*r*_*p*_ = 0.624, *p* < 0.001) and Z direction (*r*_*p*_ = 0.639, *p* < 0.001; see Figure 3). We also observed that the first-order connectivity mode in left striatum was associated with the total BPRS score (*F*(11,65) = 2.994, *p* = 0.005, *R*^*2*^ = 0.452). Post-hoc analysis revealed that *p* > 0.25 for all partial correlations of the individual TSM coefficients, suggesting that all TSM coefficients contributed more or less equally to the association. We did not observe a significant association with negative symptoms (i.e., total SANS score). Post-hoc analyses demonstrated that the observed group differences and associations with the total and positive BPRS score were not induced by age or head motion, see supplementary material.

### The cortico-striatal gradient is associated with schizotypy in the general community

**Fig. 4:**
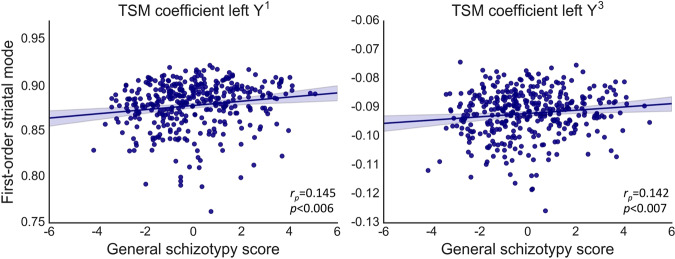
Significant association between general schizotypy and the first-order connectivity mode in left striatum. To visualize this association, partial correlations (*r*_p_) are shown between PLEs general factor score estimates and the linear and cubic TSM coefficients modeling the connectivity mode in the Y direction that most strongly contributed to the association. The shaded areas represent the confidence intervals of these correlations.

After correction for multiple comparisons, we found a weak, but significant association between the first-order connectivity mode in left striatum and inter-individual differences in the general schizotypy factor score estimates (*R*^2^ = 0.090, *F*(11,360) = 3.25, *p* < 0.001) in the community sample. Figure 4 shows the partial correlations between these factor score estimates and the TSM coefficients that most strongly contributed to the regression model. Post-hoc analyses showed that this association was not driven by age or head motion, see supplementary material. No significant associations were observed with positive or negative schizotypy factor score estimates.

## Discussion

We investigated the connectopic organization of striatum in FEP patients and in an independent community sample of non-clinical individuals. By investigating the distinct, smoothly-varying and overlapping striatal connectivity gradients obtained with connectopic mapping, we moved away from the majority of resting-state fMRI studies in psychotic disorders that have employed hard parcellations focusing on the average functional connectivity of a few discrete subregions. We investigated a gradient associated with fine-grained changes in cortico-striatal functional connectivity (first-order mode; 26) and a gradient associated with dopaminergic innervation of VTA and SN to striatum (second-order mode; 29). As hypothesized, both modes significantly differentiated FEP patients from healthy controls and were furthermore associated with positive symptoms (second-order mode) and overall symptom severity (first-order mode) in patients. In the independent sample of healthy individuals, only variations in the connectivity mode associated with cortico-striatal function were associated with inter-individual differences in the general schizotypy factor score estimate. Together, these findings suggest that altered cortico-striatal organization may be linked to the psychosis spectrum phenotype across a broad range of symptom severity, including the non-clinical range, whereas altered dopaminergic innervation, to the extent that it is captured by the second functional gradient, may be more apparent to individuals experiencing clinical levels of illness.

### The gradient associated with cortico-striatal function is disrupted in illness and associated with subclinical schizotypy

We observed that the connectivity gradient associated with cortico-striatal function changed more slowly from ventral to dorsal striatum in FEP compared to controls (Figure 2). This result might imply that in FEP patients, a larger area of the striatum is dedicated to cortico-striatal circuits typically associated with the ventral (limbic) circuit, and a smaller area to circuitry typically associated with the dorsal (cognitive/associative) circuit. This finding aligns with a large literature consistently demonstrating reduced connectivity in the dorsal cortico-striatal loop in high-risk individuals and patients [9–12, 20, 50], and with studies reporting increased connectivity in the ventral cortico-striatal loop in patients [12, 14]. Our finding might further suggest that previous results of decreased and increased connectivity in the dorsal and ventral circuit respectively, might have their basis in an altered topographic organization of striatum.

Spatial variations in this connectivity gradient were also associated with subclinical variation in the general schizotypy factor score estimate (reflecting both positive and negative aspects of PLEs and schizotypy) in our community-based sample. This result aligns with previous studies reporting that higher levels of PLEs are associated with lower functional connectivity within the dorsal [19–21] and increased connectivity within the ventral circuit [21]. Our results thus confirm that alterations in cortico-striatal connectivity are evident across the full psychosis continuum, ranging from subclinical PLEs and schizotypal traits to frank psychotic disorder. However, it should be noted that the magnitude of the associations identified here is modest and that the exact TSM coefficients in which the changes can be observed do slightly differ between the FEP and community sample. This suggests that the specific way in which this connectivity mode is implicated in clinical illness and PLEs and/or schizotypy may vary at a more fine-grained scale (i.e., at the level of the specific TSM coefficients). Whether such variations have mechanistic implications is unclear, but the results do suggest that this gradient associated with cortico-striatal function is affected in some way in both the FEP and community sample.

### Disruptions of the gradient associated with dopaminergic innervation are specific to clinical illness

The second-order striatal connectivity mode associated with dopaminergic innervation differed significantly between FEP patients and controls. Previous work in healthy controls demonstrated that this connectivity gradient shows a high spatial correspondence to DAT availability in striatum (Figure S2), a marker of dopaminergic projections. Within this connectivity mode, red voxels mapped onto high DAT availability (high density of dopaminergic projections) and blue voxels onto low DAT availability (low density of dopaminergic projections). Here we observed that FEP patients displayed changes in this connectivity mode in both the ventral (more dark blue voxels) and dorsal striatum (more dark red voxels). We speculate that these findings might suggest that dopaminergic innervation of the striatum is altered in FEP with a pattern consistent with a relative reduction of dopaminergic input to ventral striatum and an increase of dopaminergic input to dorsal striatum. PET studies in patients with psychotic disorders have repeatedly demonstrated increased levels of striatal dopamine (for review see: [51]), with several studies specifically pointing to elevated levels in dorsal striatum [4–6]. The alterations observed in the second-order connectivity mode in dorsal striatum might thus correspond with these reports of increased dopamine levels. However, it should be noted that a meta-analysis across 202 schizophrenia patients and 147 controls did not find evidence for alterations in the average DAT density across (subregions of) the striatum [52]. In addition, to our knowledge, there are no reports of decreased dopamine levels in ventral striatum. The discrepancy may reflect that the second-order mode has been shown to reliably track DAT abundance in the striatum, but studies of psychosis have focused on dopaminergic synthesis capacity, as measured using 3,4-dihydroxy-6-[18 F]-fluoro-L-phenylalanine ([18 F]DOPA). The correspondence of [18 F]DOPA levels with both pre-synaptic DAT and the second-order striatal connectivity mode requires further investigation. It should also be noted that there appears to be a small asymmetry in the NAcc region of the second-order mode in the control group, that might have contributed to the observed group difference. However, to our knowledge, there is no evidence for an asymmetry in DAT binding or dopaminergic functioning in the striatum in healthy controls (e.g., see [53]), suggesting that this could potentially represent variable signal to noise ratio in the left and right NAcc regions.

The second-order mode was not significantly associated with schizotypy in the general community. This aligns with a previous PET study indicating that healthy individuals experiencing hallucinations do not show the elevations of striatal dopamine synthesis capacity commonly reported in patients [22]. Yet, some studies have found evidence for dopaminergic alterations in people for ultra-high risk for psychosis [54–56]. As such, altered dopaminergic signaling may be present in those individuals who are transitioning to or who are already experiencing the clinical manifestations of psychosis.

Overall, our findings suggest that while cortico-striatal organization may be related to a wide spectrum of the psychosis phenotype across the non-clinical and clinical range, dopaminergic function may be more closely tied to symptom levels near the threshold for diagnosis and treatment. This hypothesis aligns with our finding that alterations in the dopaminergic connectivity mode were associated with positive symptoms in FEP patients, and prior evidence for consistent disruptions of cortico-striatal connectivity across subclinical, ARMS, and patient groups [12, 19, 50, 57].

### Limitations

While previous work has associated the first-order and second-order striatal connectivity modes to cortico-striatal connectivity and dopaminergic innervation respectively, it is not possible to directly relate alterations in these connectivity modes to underlying mechanisms based on the application of connectopic mapping alone.

Our clinical sample comprised individuals with both affective and non-affective psychosis. Past work indicates that cortico-striatal dysfunction is apparent in patients with both forms of psychotic illness [12, 58]. In line with ongoing questions about the validity of their nosological separation, we adopted a transdiagnostic approach here. Future studies in larger samples may examine whether striatal gradients are differentially affected in these two forms of psychosis.

Our community sample excluded individuals with a history of psychiatric illness or treatment, which may have restricted the observed range of symptom severity. Although similar levels of PLEs have been observed in other samples (see [19] for a discussion), the recruitment of individuals spanning a wider severity spectrum may improve power for identifying correlations between brain and behavior and shed more light on the specific point in the psychosis spectrum at which alterations in the second-order connectivity mode (associated with dopaminergic innervation) emerge.

## Conclusions

We applied a novel method to investigate fine-grained alterations of both cortico-striatal and dopaminergic organization of striatum in relation to clinical illness and subclinical schizotypy. Our results indicate that alterations of cortico-striatal connectivity are associated with both subclinical and clinical expression of psychosis, and may thus represent a neurobiological trait marker across the psychosis continuum. Disruption of the mode associated with dopaminergic innervation was only observed in patients, suggesting that dopaminergic dysfunction might be more apparent in clinical illness.

## Supplementary information

Supplementary Materials

## Data Availability

All code used for the connectopic mapping procedure is available at the following Github repository: https://github.com/koenhaak/congrads.
