# Supplementary material for: Gradients of striatal function in antipsychotic-free first-episode psychosis and schizotypy
Source: Transl Psychiatry. 2023 Apr 18;13:128. doi: 10.1038/s41398-023-02417-2 (PMC10113219; doi:10.1038/s41398-023-02417-2)
Supplement: Supplementary file 1 — Supplementary Materials [file 41398_2023_2417_MOESM1_ESM.doc]

**Supplementary Material**

**Gradients of striatal function in antipsychotic-free first-episode psychosis and schizotypy**

Marianne Oldehinkel, Jeggan Tiego, Kristina Sabaroedin, Sidhant Chopra, Shona M. Francey, Brian O’Donoghue, Vanessa Cropley, Barnaby Nelson, Jessica Graham, Lara Baldwin, Hok Pan Yuen, Kelly Allott, Mario Alvarez-Jimenez, Susy Harrigan, Christos Pantelis, Stephen J Wood, Patrick McGorry, Mark Bellgrove, Alex Fornito

Content:

**Supplementary Text 2**

Study design and participants of the community dataset. …………….……………………………………………… 2

## Measures of psychotic-like experiences (PLEs) in the community dataset. .…………………….…………… 2

Derivation of schizotypy factor score estimates using Structural Equation Modelling. 3

## Analysis of motion and age contamination. ………………………………………………………………….……………... 5

## The selection of the TSM model order and post-hoc analysis of TSM model order………………..……… 5

Supplementary Tables and Figures 7

Table S1. Descriptive statistics for the scales and subscales used to measure schizotypy in the community sample. 7

Table S2. Comparison of results for schizotypy scales in the current sample with normative samples. 8

Table S3. Schizotypy correlation matrix for the total sample. 9

Table S4. Summary of fit statistics for competing Confirmatory Factor Analysis models of schizotypy in the calibration subsample. 10

Table S5. Matrix of correlation residuals and standardized covariance residuals for the bifactor model in the calibration subsample. 11

Table S6. Invariance testing results for the bifactor schizotypy model in the community sample 12

Table S7. Number of excluded connectivity modes. 13

Table S8. Clinical characteristics of included versus excluded participants. 14

Table S9. Pearson correlations between the TSM coefficients modeling the second-order connectivity mode in left striatum.. 15

Table S10. Model fits for different TSM model orders investigating the association between the second-order connectivity mode in left striatum and the BPRS positive scores………………….………..16

Figure S1. **Bifactor model of schizotpy/psychotic-like experiences (PLEs) in the calibration subsample of the community participants.** 17

Figure S2. **The zero-order, first-order and second-order modes of connectivity in striatum**. 18

Figure S3. **The variance explained by different TSM models in the FEP dataset**. 19

Figure S4. **The variance explained by different TSM models in the community dataset**. 20

Supplementary References 21

**Supplementary Text**

*Study design and participants of the community dataset*

719 Right-handed Caucasian adults (424 females) aged 18-50 years were recruited from the general community in Melbourne and Brisbane, Australia, through local advertisements. Recruitment was part of a larger genetics study (N=1296) that required participants to have all four grandparents of European descent (1). Participants had no personal history of psychiatric illness, neurological illness, or psychotropic treatment and no intellectual disability. Additional exclusion criteria included regular use of recreational drugs for at least once a month, a history of drug abuse, and a significant blow to the head, signified by a loss of consciousness or memory. The Monash University Human Research Ethics Committee approved the study (reference number 2012001562). Each participant provided written informed consent following a thorough explanation of the study. Here we selected all the participants (N=719) that completed an online survey.

## Measures of psychotic-like experiences (PLEs) in the community dataset

A total of 719 participants completed an online survey via a personalized electronic link to Qualtrics (2), an online survey platform, which was used to administer eleven different measures of schizotypy and psychosis-like experiences (PLEs), which are described below, comprising a total of 251 items. The Peters Delusions Inventory-21 (PDI-21; 3) measures delusional ideation in the general population. The Wisconsin Schizotypy Scales (WSS) included four separate scales individually designed to measure distinct aspects of schizotypy in the general population: i) the Magical Ideation scale (30 items) measures beliefs of causation that would traditionally be viewed as invalid, such as telepathy or superstitiousness (4); ii) the Physical Anhedonia scale (40 items) measures lack of physical pleasure, such as eating, touching, sex, smell, and sound (5); iii) The Revised Social Anhedonia scale (40 items) – measures a lack of pleasure in interpersonal activities (Eckblad et al. unpublished questionnaire, 1982); iv) The Perceptual Aberration scale (35 items) – measures perceptual distortions, including of the physical body, sight, and hearing (6). The Community Assessment of Psychic Experience (CAPE) is a self-report questionnaire, measuring psychotic-like experiences in the general population (7). The CAPE consists of 42 items across three symptom dimensions: 1) Positive (20 items); 2) Negative (14 items); and 3) Depressive (8 items). The Oxford-Liverpool Inventory of Feelings and Experiences Short Form (sO-LIFE; 8) is a self-report questionnaire designed to incorporate the main aspects of schizophrenia-like experiences using four subscales: 1) Unusual Experiences (12 items), consistent with positive symptoms; 2) Cognitive Disorganization (11 items), including difficulties in cognition; 3) Introvertive Anhedonia (10 items), tapping into a lack of social enjoyment; and 4) Impulsive Nonconformity (10 items), incorporating violent and reckless behaviors. For all these questionnaires higher scores indicated higher levels of schizotypy/PLEs.

*Derivation of schizotypy factor score estimates using Structural Equation Modelling*

We used structural equation modeling (9) to characterize the latent structure of schizotypy. To this end, item-level response data from these subjects was downloaded directly from Qualtrics as an SPSS data file and data screening and preliminary analyses were conducted in IBM SPSS Statistics Version 27 (10). Confirmatory Factor Analysis (CFA) models were subsequently estimated in Mplus 8.3 based on the covariance matrix of continuous data for the questionnaire subscales using full information maximum likelihood to account for missing data and the Bollen-Stine Bootstrap procedure with 10,000 posterior draws (11, 12). Factor scaling was performed using the reference variable method (13). *Post hoc* model fitting was performed by freeing cross-loadings and error covariances for estimation one at a time with reference to theoretical plausibility and modification indices and corrected for significance using the Benjamini-Hochberg (B-H) False Discovery Rate (FDR, q = .05; 13, 14, 15).

Model fit was evaluated using a combination of fit indices. The chi square (χ^2^) test statistic is the gold standard index of global fit, with higher probability values signifying greater probability of the null hypothesis of exact fit of the model reproduced covariance matrix to the observed covariance matrix (16, 17). We also report three approximate fit indices - the root mean square error of approximation (RMSEA) and associated 90% confidence interval (90%*CI*), the comparative fit index (CFI), and standardized root mean square residual (SRMR; 18, 19). Higher values for the CFI and lower values of the RMSEA and SRMR are indicative of better fitting models (17, 20). We also inspected and report the correlation residuals (ε) and standardized covariance residuals (*Z* scores), which represent absolute differences between expected and model reproduced correlations / covariances. A pattern of correlation residuals exceeding the recommended threshold of >0.10, or covariance residuals exceeding 1.96, *p*<0.05 may be indicative of poor local fit (21). A competing models strategy was used, which involved estimating several alternative models and comparing them in terms of data fit (22). Statistically non-nested models were compared using Bayesian statistics, specifically the Bayesian conditional posterior probability, which is derived from the Bayesian Information Criterion ((BIC), [PrBIC(Hi|D)]; 23, 24, 25).

The participant sample was randomly split into two approximately equal groups: a calibration subsample (*N*=359; 217 females) aged 18–47 years (*M*=23.1, *SD*=4.7), and a validation subsample (*N*=360; 204 females), aged 18–48 years (*M*=23.4, *SD*=4.9). There were no statistically significant differences in the sex (*χ*^2^(1)=.806, *p*=.369) or age (*t*(714)=-.824, *p*=.410; 95%*CI*=-1.007–.412) composition of the subsamples. All CFA models were first tested in the calibration subsample and then cross-validated in the validation subsample using invariance testing (13). Invariance testing was performed using the free-baseline approach, which entails fewer assumptions and proceeds from testing the least restrictive to increasingly strict equality constraints on model parameters (26, 27). We tested for invariance in the following order: 1) configural invariance – same pattern of factor loadings; 2) weak invariance – statistical equivalence of unstandardized factor loading estimates; 3) strong invariance – statistical equivalence of intercepts; 4) strict invariance – equality of unstandardized error variances and covariances; and 5) structural invariance – equality of factor variances and covariances (28). Partial invariance was considered where full invariance did not apply (19). We used a backwards sequential search strategy (29), by freeing parameters for estimation one at a time in the AMT validation subsample and community and clinical sample based on inspection of between-group differences in unstandardised estimates. The backwards sequential search method has been shown to have good sensitivity for detecting true positives while also yielding a low rate of false positives (i.e. Type I error control; 30). Invariance was tested using the chi square difference test (Δχ^2^) and a designated *p* value threshold of *p*<.05 (16). The Δχ^2^ is well-powered to detect differences between groups when sample size is large.

Descriptive statistics for the PLEs/schizotypy questionnaires can be found in Table S1 and comparison of the results to those reported for normative samples are reported in Table S2. The correlation matrix for the subscale scores is provided in Table S3. Fit statistics for the competing models are displayed in Table S4. The best-fitting model was a bifactor model with a General schizotypy factor with primary loadings from all subscales except for Physical Anhedonia, and two group factors capturing residual variance specific to positive and negative schizotypy (χ^2^(46)=58.794, *p*=.264; RMSEA=.028 [95%CI=.000, .047]; CFI=.994; SRMR=.031). This model is displayed in Figure S1 and provided an excellent fit to the data as observed in the indices of global fit, as well as the matrix of residuals, where none of the 78 correlation residuals exceeded .10 and only 1/77 estimated covariance residuals exceeded the critical probability threshold after correction for multiple comparisons (see Table S5). Cross validation in the hold-out sample provided evidence that the model exhibited close to full invariance across all estimated parameters (see Table S6), except for a statistically significant difference in the strength of the unstandardized loading of the CAPE Depressive subscale on the general schizotypy factor, which is likely to have minimal impact on the measurement properties of this factor. This indicates that the measurement properties of the bifactor model were very stable within sample. Factor score determinacies, which correspond to validity coefficients, were high for the general, positive and negative schizotypy factors (ρ=.902, .901, .913), indicating that the factor score estimates provided highly accurate measures of individual differences on the three latent continuums (31). Furthermore, the factor score estimates were only very weakly correlated (*r*=-.075–.147), indicating that the orthogonality of the three factors in the model had been largely preserved and that there was minimal measurement contamination from other factors when generating the factor score estimates for each of the three schizotypy factors.

*Analysis of motion and age contamination*

In addition to our stringent quality control procedures, we conducted additional post-hoc analyses to test for any residual effects of head motion and effects of age on our findings described in the manuscript. For all significant group differences and associations observed in this study, we conducted post-hoc analyses to demonstrate that these findings were not induced by age or head motion. First, meanFD was added as a nuisance covariate to the binary logistic regression or GLM (age was already included in the main analysis). Second, correlations were computed between meanFD as well as DVARS and the TSM coefficients, and between age and the TSM coefficients. None of the associations of age, meanFD and DVARS with the TSM coefficients were significant (FEP dataset: all abs(*r*)<0.211, all p>0.057; community dataset: all abs(*r*)<0.094, all p>0.067).

We furthermore computed correlations between the psychosis measures in the FEP dataset and meanFD and DVARS to make sure that meanFD and DVARS are not correlated to symptom severity (correlations with meanFD: BPRS total score: *r*=0.089, *p*=0.517; BPRS positive score: *r*=-0.138, *p*=0.217; SANS total score: *r*=-0.065, *p*=0.563; correlations with DVARS: BPRS total score *r*=0.121, *p*=0.374; BPRS positive score: *r*=0.028, *p*=0.803; SANS total score: *r*=0.072, *p*=0.519). Similarly, meanFD was not correlated to the schizotypy measures in the community dataset (correlation of meanFD with general schizotypy factor score estimate: *r*=-0.015, *p*=0.774; positive schizotypy factor score estimate: *r*=0.092, *p*=0.074; and negative schizotypy factor score estimate: *r*=0.058, *p*=0.260).

*The selection of TSM model order and post-hoc analyses of TSM model order*

The last step in the connectopic mapping analysis pipeline consists of selecting the model order of the TSM model that best describes each of the connectivity modes. This choice is based on how well the TSM model fits the individual subject gradients and is thus independent of the specific analysis conducted. To select the degree of the interpolating polynomial basis set, we fitted these models across polynomials of degrees ranging from 1 to 5 and then compared the different model orders using a Scree plot analysis (49). Scree-plot analysis revealed, in line with previous work, that a third-degree polynomial model (including 9 TSM) coefficients, provided the best fit for each of the investigated connectivity modes in both the FEP dataset and community dataset, see Supplementary Figures S3 and S4.

However, while a third-order polynomial TSM model might be the best fit based on explained variance of the individual subjects’ connectivity modes, one might run the risk of overfitting when applying this model to a sample with a low number of subjects. While we are confident that the observed association between the TSM coefficients modeling the second-order connectivity mode in left striatum and BPRS positive symptoms shown in Figure 3 is not the result of overfitting, we nevertheless ran a few additional analyses to demonstrate that our TSM models are not overfitting the data and the TSM coefficients are not collinear. To this end, we first computed Pearson correlations between the TSM coefficients modeling the second-order connectivity mode in left striatum (as shown in Figure 3). From these correlations that are listed in Table S9, it can be observed that there are relatively high correlations (*r*>0.7) between the linear and cubic coefficients, which are highlighted in bold. To demonstrate that the significant association between the second-order connectivity mode and BPRS positive symptom scores was not induced by collinear coefficients, we removed all the cubic coefficients, leaving us with the linear and quadratic TSM coefficients alone, and then repeated our main analysis with these remaining 6 TSM coefficients. The GLM now revealed a trend-level association (*F*(8,51)=1.987 *p*=0.072, *R^2^*=0.319) and the partial correlations of the quadratic TSM coefficients with the BPRS positive scores were also still significant in the X (*r_p_*=0.397, *p*=0.017), Y (*r_p_*=0.396, *p*=0.017) and Z direction (*r_p_*=0.428, *p*=0.009). Similarly, we also repeated the analysis by excluding the linear coefficients, while keeping the quadratic and cubic coefficients in the GLM model. This revealed both a significant association (*F*(8,51)=2.230 *p*=0.041, *R^2^*=0.354) and significant partial correlations between the quadratic TSM coefficients and the BPRS positive scores in the X (*r_p_*=0.446, *p*=0.006), Y (*r_p_*=0.448, *p*=0.006) and Z direction (*r_p_*=0.452, *p*=0.006). Although the above associations and the partial correlations are less strong –which is not surprising given that previous work showed a model with 9 (and not 6) TSM coefficients was optimal and fewer TSM coefficients (6 vs 9) were included in the GLM– these results do demonstrate that the association with BPRS positive symptom scores was not induced by collinear coefficients and furthermore that the association with the BPRS positive score can be replicated using a TSM model of a lower model order.

Finally, we also conducted an additional analysis in which we compute several metrics to define the most appropriate TSM model to use for this specific multiple linear regression analysis associating TSM coefficients to BPRS positive symptoms. Supplementary Table S10 lists the coefficient of determination, or *R^2^* square (variance explained by the model), the adjusted *R^2^* (penalizing for the additional TSM parameters) and the Bayesian Information Criterion (BIC) for polynomial TSM models with model orders ranging from 1 to 5. All three criteria strongly favor a third-order polynomial TSM model (model order 3) over the other model orders. When inspecting the *R^2^* and adjusted *R^2^*, both values substantially increase form model order 2 to model order 3, but not that much anymore from model order 4 onwards. Similarly, the BIC value for model order 3 is lowest, indicating the best fit. Accordingly, these results indicate that a TSM model with a polynomial degree of 3 also provides the best fit for this specific analysis investigating the association between the TSM coefficients and BPRS positive symptom scores.

# Supplementary Tables and Figures

**Table S1. Descriptive statistics for the scales and subscales used to measure schizotypy in the community sample.**

| Variable  Variable | *M* | 95% *CI*  *LL UL* | | *Median* | *SD* | *Range* | *Skewness*^1^ | *Kurtosis^2^* | *Reliability*^3^ | *N* |
| --- | --- | --- | --- | --- | --- | --- | --- | --- | --- | --- |
| PDI-21 | 5.16 | 4.82 | 5.50 | 5 | 3.32 | 0 - 14 | 4.87^***^ | -.385 | .77 | 375 |
| WSS |  |  |  |  |  |  |  |  |  |  |
| Magical | 5.93 | 5.48 | 6.37 | 5 | 4.41 | 0 - 22 | 7.16^***^ | 1.88 | .83 | 376 |
| Perceptual | 4.83 | 4.36 | 5.31 | 3 | 4.63 | 0 - 20 | 19.89^***^ | 4.13^***^ | .88 | 370 |
| Social | 9.45 | 8.81 | 10.10 | 8 | 6.339 | 0 - 32 | 8.08^***^ | 4.27^***^ | .86 | 372 |
| Physical | 11.49 | 10.80 | 12.17 | 10 | 6.715 | 0 - 33 | 5.99^***^ | 8.27^***^ | .85 | 374 |
| CAPE-42 |  |  |  |  |  |  |  |  |  |  |
| Positive | 1.27 | 1.25 | 1.29 | 1.2 | 0.20 | 1.0–1.9 | 7.59^***^ | 2.21^*^ | .80 | 360 |
| Negative | 1.71 | 1.66 | 1.75 | 1.64 | 0.41 | 1.0–3.0 | 5.31^***^ | 0.84 | .81 | 365 |
| Depressive | 1.71 | 1.67 | 1.74 | 1.63 | 0.34 | 1.0–2.6 | 4.42^***^ | 0.03 | .80 | 359 |
| *s*O-LIFE |  |  |  |  |  |  |  |  |  |  |
| Unusual | 2.81 | 2.55 | 3.07 | 2 | 2.55 | 0 - 10 | 7.21^***^ | 0.82 | .78 | 364 |
| Cognitive | 4.68 | 4.38 | 4.98 | 5 | 2.92 | 0 - 11 | 1.26 | -3.16^**^ | .76 | 366 |
| Impulsive | 3.18 | 2.96 | 3.39 | 3 | 2.080 | 0 - 10 | 3.33^***^ | -1.64 | .60 | 366 |
| Introvertive | 1.57 | 1.41 | 1.72 | 1 | 1.53 | 0 - 6 | 7.76^***^ | 1.87 | .60 | 359 |

*M =* Mean; *CI* = Confidence Interval; *LL* = Lower Limit; *UL* = Upper Limit; *SD* = Standard Deviation; *N* = size of subsample with non-missing data. ^1^ *z* score calculated by dividing the skewness statistic by its standard error. ^2^ *z* score calculated by dividing the skewness statistic by its standard error. ^3^ Internal consistency reliability calculated as Cronbach’s alpha (α) in all available date from the full sample (*N*=727). WSS = Wisconsin schizotypy scales; Magical = Magical ideation scale; Perceptual = Perceptual aberration scale; Social = Social anhedonia scale; Physical = Physical anhedonia scale; CAPE-42 = Community Assessment of Psychic Experiences 42-item version; Positive = CAPE Positive symptoms subscale; Negative = CAPE Negative symptoms subscale; Depressive = CAPE Depressive symptoms subscale; *s*O-LIFE = Oxford-Liverpool Inventory of Feelings and Experiences short-form; Unusual = *s*O-LIFE Unusual Experiences subscale; Cognitive = *s*O-LIFE Cognitive Disorganization subscale; Impulsive = *s*O-LIFE Impulsive Non-Conformity subscale; Introvertive = *s*O-LIFE Introvertive Anhedonia subscale. *** *p*<.001 ** *p*<.01. * *p*<.05.

## Table S2. Comparison of results for schizotypy scales in the current sample with normative samples.

|  | Females | | | | | | | Males | | | | | | |
| --- | --- | --- | --- | --- | --- | --- | --- | --- | --- | --- | --- | --- | --- | --- |
| Variable | Δ*M* | *SE* | 95%*CI* | *df* | *t* | *p* | *d* | Δ*M* | *SE* | 95%*CI* | *df* | *t* | *p* | *d* |
| PDI-21^1^ | 1.58 | 0.37 | 0.87, 2.30 | 468 | 4.34 | <.001 | .41 | 1.31 | 0.42 | 0.50, 2.13 | 347 | 3.16 | .002 | .34 |
| *s*O-LIFE^2^ |  |  |  |  |  |  |  |  |  |  |  |  |  |  |
| Unusual | 0.35 | 0.22 | -0.08, 0.79 | 1733 | 1.61 | .108 | .12 | 0.67 | 0.27 | 0.13, 1.20 | 419 | 2.46 | .015 | .25 |
| Cognitive Disorganization | -0.44 | 0.21 | -0.86, 0.02 | 1734 | -2.05 | .041 | .15 | -0.15 | 0.30 | -0.73, 0.44 | 420 | -0.49 | .626 | .05 |
| Impulsive | -0.63 | 0.15 | -0.92, -0.34 | 1734 | -4.24 | <.001 | .31 | -0.43 | 0.20 | -0.83, -0.02 | 420 | -2.08 | .038 | .21 |
| Introvertive | 0.96 | 0.14 | 0.68, 1.24 | 1732 | 6.74 | <.001 | .57 | 1.07 | 0.20 | 0.67, 1.46 | 415 | 5.24 | <.001 | .55 |
| WSS^3^ |  |  |  |  |  |  |  |  |  |  |  |  |  |  |
| Magical Ideation | 2.95 | 0.40 | 2.16, 3.74 | 3566 | 7.34 | <.001 | .57 | 3.61 | 0.46 | 2.71, 4.50 | 1335 | 7.91 | <.001 | .73 |
| Physical Anhedonia | 0.13 | 0.42 | -0.71, 0.96 | 3566 | 0.30 | .766 | .02 | 1.67 | 0.65 | 0.40, 2.94 | 1333 | 2.58 | .010 | .22 |
| Perceptual Aberration | 0.67 | 0.40 | -0.13, 1.45 | 3562 | 1.65 | .099 | .12 | 1.31 | 0.47 | 0.39, 2.23 | 1333 | 2.80 | .005 | .26 |
| Social Anhedonia | 3.54 | 0.40 | 2.76, 4.33 | 3565 | 8.86 | <.001 | .66 | -1.02 | 0.54 | -2.07, 0.03 | 1332 | -1.91 | .056 | .15 |
| CAPE^4^ | Females and Males | | | | | | |  |  |  |  |  |  |  |
| Positive | 0.33 | 0.02 | 0.30, 0.37 | 2633 | 20.4 | <.001 | 1.31 |  |  |  |  |  |  |  |
| Negative | 0.09 | 0.02 | 0.05, 0.14 | 2638 | 4.14 | <.001 | .23 |  |  |  |  |  |  |  |
| Depressive | 0.29 | 0.02 | 0.25, 0.34 | 2632 | 13.1 | <.001 | .79 |  |  |  |  |  |  |  |

Δ*M* = *M*_normative sample_ - *M*_study sample_.  *SE* = standard error of the mean difference. 95%*CI* = 95% confidence interval for the mean difference. *df* = degrees of freedom; *t* = *t* test statistic; *p* = probability value of the *t* test statistic; *d* = Cohen’s *d*. ^1^ (32); ^2^ (33); ^3^ (34); ^4^ (35). Separate normative data were not available for female and male participants. Therefore, statistics reflect comparisons between the combined samples.

## Table S3. Schizotypy correlation matrix for the total sample.

| Variable | 1. | 2. | 3. | 4. | 5. | 6. | 7. | 8. | 9. | 10. | 11. | 12. |
| --- | --- | --- | --- | --- | --- | --- | --- | --- | --- | --- | --- | --- |
| 1. Age in Years |  |  |  |  |  |  |  |  |  |  |  |  |
| 2. PDI | -.100  [-.172, -.037] |  |  |  |  |  |  |  |  |  |  |  |
| 3. Magical Ideation | -.092  [-.161, -.017] | .679  [.649, .734] |  |  |  |  |  |  |  |  |  |  |
| 4. Unusual Experiences | -.098  [-.170, -.027] | .625  [.584, .687] | .729  [.698, .773] |  |  |  |  |  |  |  |  |  |
| 5. CAPE Positive | -.065  [-.140, .000] | .811  [.792, .848] | .719  [.687, .763] | .702  [.671, .748] |  |  |  |  |  |  |  |  |
| 6. Perceptual Aberration | -.143  [-.211, -.079] | .566  [.541, .645] | .613  [.576, .679] | .672  [.639, .729] | .601  [.554, .665] |  |  |  |  |  |  |  |
| 7. Impulsive Non-Conformity | -.114  [-.182, -.040] | .431  [.383, .512] | .471  [.422, .543] | .522  [.478, .595] | .461  [.409, .534] | .409  [.345, .486] |  |  |  |  |  |  |
| 8. Cognitive Disorganization | -.168  [-.237, -.089] | .366  [.303, .440] | .346  [.284, .423] | .432  [.373, .500] | .305  [.245, .375] | .333  [.278, .411] | .396  [.341, .468] |  |  |  |  |  |
| 9. CAPE Negative | -.146  [-.216, -.067] | .376  [.325, .456] | .275  [.212, .354] | .341  [.283, .418] | .346  [.283, .419] | .366  [.308, .440] | .278  [.215, .353] | .519  [.473, .575] |  |  |  |  |
| 10. Physical Anhedonia | -0.081  [-.150, -.005] | -.070  [-.147, -.002] | -.079  [-.154, -.007] | -.043  [-.123, .024] | -.068  [-.143, -.001] | -.013  [-.091, .057] | -.015  [-.093, .052] | .146  [.068, .210] | .311  [.238, .372] |  |  |  |
| 11. Social Anhedonia | .046  [-.023, .120] | .136  [.086, .227] | .130  [.066, .202] | .202  [.139, .274] | .163  [.098, .240] | .220  [.146, .284] | .190  [.112, .262] | .311  [.239, .376] | .511  [.452, .563] | .438  [.385, .510] |  |  |
| 12. Introvertive Anhedonia | 0.019  [-.062, .095] | .122  [.053, .215] | .079  [.014, .167] | .145  [.086, .232] | .055  [-.005, .152] | .116  [.059, .202] | .146  [.090, .243] | .341  [.304, .431] | .439  [.398, .520] | .516  [.459, .574] | .649  [.605, .695] |  |
| 13. CAPE Depressive | -0.144  [-.214, -.064] | .457  [.430, .538] | .354  [.309, .442] | .392  [.351, .479] | .416  [.373, .495] | .383  [.321, .448] | .397  [.349, .464] | .533  [.486, .589] | .552  [.494, .604] | .092  [-.011, .143] | .219  [.141, .284] | .245  [.163, .310] |

Correlations with bootstrapped (10,000 posterior draws) 95% confidence intervals displayed in brackets. *N*=719.

## Table S4. Summary of fit statistics for competing Confirmatory Factor Analysis models of schizotypy in the calibration subsample

|  | Model | *df* | *χ*^2^ | *p* | RMSEA (90%*CI*) | CFI | SRMR | BIC | Pr (*H_i_* | *D*) |
| --- | --- | --- | --- | --- | --- | --- | --- | --- | --- |
| 1 | One-Factor^1^ | 52 | 143.536 | <.001 | .070 (.057, .084) | .958 | .053 | 22370.016 | <.001 |
| 2 | Positive & Negative Schizotypy  Correlated Two-Factor^2^ | 53 | 190.261 | <.001 | .085 (.072, .098) | .936 | .088 | 22410.858 | <.001 |
| 3 | Positive, Negative, & Disorganized Schizotypy  Correlated Three-Factor^3^ | 52 | 126.520 | <.001 | .063 (.049, .077) | .966 | .046 | 22353.000 | <.001 |
| 4 | Bi-Factor Model^4^* | 46 | 58.794 | .264 | .028 (.000, .047) | .994 | .031 | 22320.574 | >.999 |

*df* = Degrees of Freedom; *χ*^2^ = Chi square value for test of model fit using maximum likelihood estimation with expectation maximisation; *p =* significance value of the *χ*^2^ test statistic with Bollen-Stine bootstrapping (10,000 posterior draws); RMSEA = Root Mean Square Error of Approximation; *CI* = Confidence Interval; SRMR = Standardised Root Mean Residual; CFI = Comparative Fit Index; BIC = Bayesian information criterion; Pr (*H_i_* | *D*) = Bayesian conditional posterior probability of model compared to all models tested. *N*=359. ^1^ Post hoc model modifications included thirteen error covariances, all significant when corrected for multiple comparisons (B-H *p*=.003). ^2^ Post hoc model modifications included two freely estimated cross-loadings and eight error covariances, all significant when corrected for multiple comparisons (B-H *p*=.005). ^3^ Post hoc model modifications included a freely estimated cross-loading and six error covariances, all significant when corrected for multiple comparisons (B-H *p*=.002). ^4^ Post hoc model modifications included eight error covariances, all significant when corrected for multiple comparisons (B-H *p*=.029). * Preferred model based on comparison of fit statistics.

## Table S5. Matrix of correlation residuals and standardized covariance residuals for the bifactor model in the calibration subsample.

| Variable | 1. | 2. | 3. | 4. | 5. | 6. | 7. | 8. | 9. | 10. | 11. | 12. | 13. |
| --- | --- | --- | --- | --- | --- | --- | --- | --- | --- | --- | --- | --- | --- |
| 1. Age in Years |  | -.743  [.457] | -.248  [.804] | -.502  [.616] | -.570  [.569] | -1.595  [.111] | .150  [.881] | .619  [.536] | .473  [.636] | 2.162  [.031] | 2.171  [.030] | 2.204  [.028] | .889  [.374] |
| 2. PDI | -.027 |  | .497  [.619] | -1.342  [.180] | -.554  [.580] | .795  [.427] | -1.160  [.246] | -1.202  [.229] | -.081  [.935] | -.951  [.342] | -.430  [.667] | -1.381  [.167] | 1.101  [.271] |
| 3. Magical Ideation | .067 | .003 |  | -.999 | -.058  [.954] | .242  [.809] | -.492  [.623] | .949  [.343] | -1.537  [.124] | -1.524  [.128] | -1.006  [.314] | -1.732  [.083] | -.448  [.654] |
| 4. Unusual Experiences | - .019 | -.013 | .001 |  | -3.464*  [<.001] | .017  [.986] | 2.003  [.045] | .781  [.435] | -.442  [.658] | -.386  [.699] | .582  [.561] | -.461  [.645] | -2.264  [.024] |
| 5. CAPE Positive | -.022 | -.002 | .001 | -.006 |  | -1.385  [.166] | .540  [.589] | -1.593  [.111] | -1.013  [.311] | -1.385  [.166] | -.575  [.565] | -1.880  [.060] | 1.014  [.311] |
| 6. Perceptual Aberration | .067 | .013 | -.001 | -.001 | .009 |  | -.732  [.464] | -.458  [.647] | .017  [.986] | .043  [.966] | 1.594  [.111] | -.345  [.731] | -1.690  [.091] |
| 7. Impulsive Non-Conformity | .005 | -.025 | -.004 | .050 | .012 | -.021 |  | .798  [.425] | -.018  [.986] | -.570  [.569] | .524  [.600] | .029  [.977] | -1.785  [.074] |
| 8. Cognitive Disorganization | .019 | -.027 | .024 | .019 | -.035 | -.012 | .017 |  | -3.089  [.002] | -.283  [.777] | -.335  [.738] | .705  [.481] | 2.168  [.030] |
| 9. CAPE Negative | .014 | .001 | -.038 | -.042 | -.018 | .043 | .002 | -.042 |  | -.735  [.462] | -.521  [.602] | -.980  [.327] | .787  [.431] |
| 10. Physical Anhedonia | .067 | -.050 | -.081 | -.021 | -.067 | .002 | -.031 | -.010 | -.018 |  | -2.225  [.026] | -2.054  [.040] | .289  [.772] |
| 11. Social Anhedonia | .102 | -.014 | -.041 | .024 | -.023 | .067 | .022 | -.006 | -.003 | -.016 |  | -.709  [.478] | -1.023  [.306] |
| 12. Introvertive Anhedonia | .107 | -.057 | -.075 | -.019 | -.080 | -.015 | .001 | .017 | -.014 | -.014 | -.008 |  | .240  [.810] |
| 13. CAPE Depressive | .027 | .026 | -.011 | -.049 | .025 | -.045 | -.037 | .037 | .023 | .016 | -.037 | .010 |  |

Lower diagonal contains the correlation residuals. Upper diagonal contains the standardized covariance residuals (i.e., Z values), with corresponding two-tailed *p* values in brackets below. -.999 estimate unavailable. *N*=359. ^*^ B-H *p*=.005.

## Table S6. Invariance testing results for the bifactor schizotypy model in the community sample.

| Model | *df* | *χ*^2^ | *p* | RMSEA [90%*CI*] | CFI | SRMR | Δ*df* | Δ*χ*^2^ | Δ*p* |
| --- | --- | --- | --- | --- | --- | --- | --- | --- | --- |
| Configural Invariance^1^ | 94 | 131.069 | .080 | .033 [.018, .046] | .991 | .034 |  |  |  |
| Partial Weak Invariance^2^ | 112 | 156.210 | .078 | .033 [.019, .045] | .989 | .038 | 18 | 25.141 | .121 |
| Strong Invariance | 125 | 164.138 | .132 | .030 [.015, .041] | .991 | .039 | 13 | 7.928 | .848 |
| Strict Invariance | 141 | 186.173 | .138 | .030 [.016, .041] | .989 | .042 | 16 | 22.035 | .142 |
| Invariance of Factor Variances & Covariances | 146 | 194.342 | .110 | .030 [.018, .041] | .988 | .051 | 5 | 8.169 | .147 |

*df* = Degrees of Freedom; *χ*^2^ = Chi square value for test of model fit using maximum likelihood estimation with expectation maximization; *p =* significance value of the chi square test statistic with Bollen-Stine bootstrapping (10,000 posterior draws); RMSEA = Root Mean Square Error of Approximation; *CI* = Confidence Interval; SRMR = Standardised Root Mean Square Residual; CFI = Comparative Fit Index; Δ*df* = delta degrees of freedom; Δ*χ*^2^ = delta chi square; Δ*p =* significance value of the delta chi square test statistic. Calibration *N*=359. Validation subsample *N*=360. ^1^ Model in the validation subsample included eight freely estimated error covariances, all significant when corrected for multiple comparisons using the Benjamini-Hochberg false discovery rate (B-H *p*=.034). ^2^ Unstandardized loading of CAPE Depressive on the general Schizotypy factor was freely estimated in the validation subsample.

## Table S7. Number of excluded connectivity modes.

| **Connectivity mode** | **Exclusions FEP Patients**  *(Total: N=56)* | **Exclusions FEP controls**  *(Total: N=27)* | **Exclusions Schizotypy dataset**  *(Total: N=377)* |
| --- | --- | --- | --- |
| First-order mode left striatum | 2 | 3 | 1 |
| First-order mode right striatum | 4 | 0 | 2 |
| Second-order mode left striatum | 13 | 7 | 38 |
| Second-order mode right striatum | 15 | 5 | 33 |

The number of connectivity modes that were excluded from the first-episode psychosis (FEP) dataset and the schizotypy dataset due to poor quality.

## Table S8. Sample characteristics of included vs excluded patients of the FEP dataset.

|  | **Included**  **Psychosis patients**  **(N=38)** | | **Excluded**  **Psychosis patients**  **(N=18) ^a^** | | **T / χ^2^** ^b^ | ***p*** ^c^ |
| --- | --- | --- | --- | --- | --- | --- |
| Baseline age, years *(SD)* | 19.4 | *2.95* | 18.6 | *2.78* | 0.92 | *0.363* |
| Females, N *(%)* | 20 | *52.6* | 10 | *55.6%* | 0.04 | *0.838* |
| Handedness, left, N *(%)* | 1 | *2.6%* | 3 | *16.7%* | 3.63 | *0.057* |
| Education, years *(SD)* | 12.2 | *2.16* | 12.2 | *2.20* | 0.07 | *0.944* |
| Head motion, meanFD *(SD)* | 0.044 | *0.016* | 0.068 | *0.066* | **-1.56** | ***0.035*** |
| Diagnosis, N  Major depression with psychosis  Schizophreniform disorder  Psychotic disorder NOS  Substance-induced psychotic disorder  Delusional disorder  Schizophrenia  Missing diagnosis | 7  6  10  1  4  9  1 |  | 4  2  5  4  1  2  0 |  |  |  |
| BPRS total score, mean *(SD)* | 56.4 | *9.94* | 59.1 | *10.5* | -0.93 | *0.357* |
| BPRS positive subscale score, mean *(SD)* | 15.2 | *3.72* | 18.1 | *4.92* | **-2.38** | ***0.021*** |
| SANS total score, mean *(SD)* | 33.5 | *18.2* | 37.0 | *15.65* | -0.70 | *0.489* |

Abbreviations: meanFD = mean framewise displacement; NOS = not otherwise specified; BPRS = Brief Psychiatric Rating Scale version 4; SANS = Scale for Assessment of Negative Symptoms^. a^ The number of excluded participants depended on the connectivity mode (i.e., left or right, first-order or second-order mode). Here we combined all patients excluded for one or more connectivity mode analyses into one group and compared this ‘excluded’ group with patients that were included for the analyses of all the connectivity modes. ^b^ This column provides the T or χ^2^ values comparing included versus excluded participants; ^c^ and the corresponding *p*-value is shown in the last column.

**Table S9. Pearson correlations between the TSM coefficients modeling the second-order connectivity mode in left striatum.**

|  | Left x^1^ | left y^1^ | left z^1^ | left x^2^ | left y^2^ | left z^2^ | left x^3^ | left y^3^ | left z^3^ |
| --- | --- | --- | --- | --- | --- | --- | --- | --- | --- |
| left x^1^ | 1 | -.572 | -.444 | .064 | .381 | -.114 | **-.944** | .325 | .182 |
| left y^1^ | -.572 | 1 | .273 | -.224 | -.093 | -.092 | .532 | **-.749** | -.258 |
| left z^1^ | -.444 | .273 | 1 | -.119 | -.397 | .570 | .485 | -.370 | **-.829** |
| left x^2^ | .064 | -.224 | -.119 | 1 | -.395 | -.259 | -.123 | .302 | .271 |
| left y^2^ | .381 | -.093 | -.397 | -.395 | 1 | -.662 | -.324 | .329 | .184 |
| left z^2^ | -.114 | -.092 | .570 | -.259 | -.662 | 1 | .157 | -.301 | -.553 |
| left x^3^ | **-.944** | .532 | .485 | -.123 | -.324 | .157 | 1 | -.280 | -.277 |
| left y^3^ | .325 | **-.749** | -.370 | .302 | .329 | -.301 | -.280 | 1 | .440 |
| left z^3^ | .182 | -.258 | **-.829** | .271 | .184 | -.553 | -.277 | .440 | 1 |

Pearson correlations between the TSM coefficients modeling the second-order connectivity mode in left striatum as shown in Figure 3.

**Table S10. Model fits for different TSM model orders investigating the association between the second-order connectivity mode in the left striatum and the BPRS positive scores.**

|  | mo1 | mo2 | mo3 | mo4 | mo5 |
| --- | --- | --- | --- | --- | --- |
| R^2^ (adjusted R^2^) | 0.224 *(0.130)* | 0.300 *(0.137)* | 0.616 *(0.474)* | 0.626 *(0.423)* | 0.677 *(0.430)* |
| BIC | 43.508 | 45.529 | 41.375 | 45.681 | 48.012 |

Numbers in this table indicate for each model order (mo; ranging from 1 to 5) the R square (R^2^), the adjusted R^2^, and the Bayesian Information Criterion (BIC) for the multiple linear regression model investigating the association between the TSM coefficients modeling the second-order connectivity mode in the left striatum and the BPRS positive scores depicted in Figure 3.





Figure S1. Bifactor model of schizotypy/psychotic-like experiences (PLEs) in the calibration subsample of the community participants. Fit statistics were (χ^2^(46)=58.794, *p*=.264; RMSEA=.028 [90%*CI*=.000, .047]; CFI=.994; SRMR=.031). *N*=359. Error covariances all significant when corrected for multiple comparisons using the Benjamini-Hochberg false discovery rate (B-H *p*=.029). Latent correlation between Physical Anhedonia error variance and Age (Ψ=-.155, *SE*=.052, *p*=.002) omitted for clarity.

**
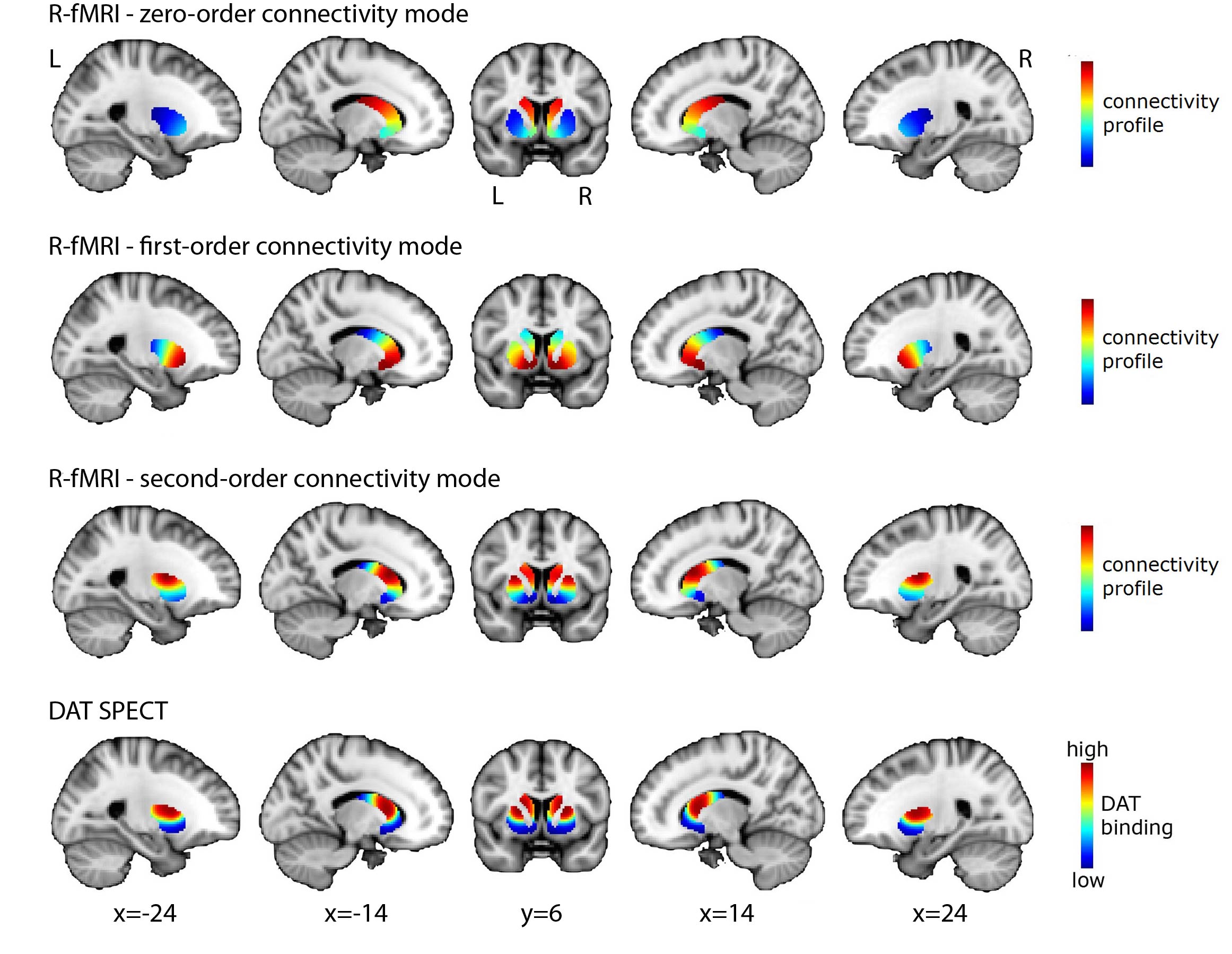
**

Figure S2. The zero-order, first-order and second-order modes of connectivity in striatum. The figure displays the group-level connectivity modes obtained in 839 Human Connectopic Project (HCP) subjects, as described by (36)**.** The zero-order mode reflects the anatomical subdivision in striatum into putamen, caudate and nucleus accumbens, whereas the first-order connectivity mode maps onto cortico-striatal connectivity (37). The second-order connectivity mode was found to map onto dopaminergic projections to striatum, as revealed by its very high spatial correlation (*r*=.884) with a group-average DAT SPECT image, a marker of dopaminergic projections. For more details see (36). Abbreviations: L = left, R = right, R-fMRI = resting-state functional magnetic resonance imaging.


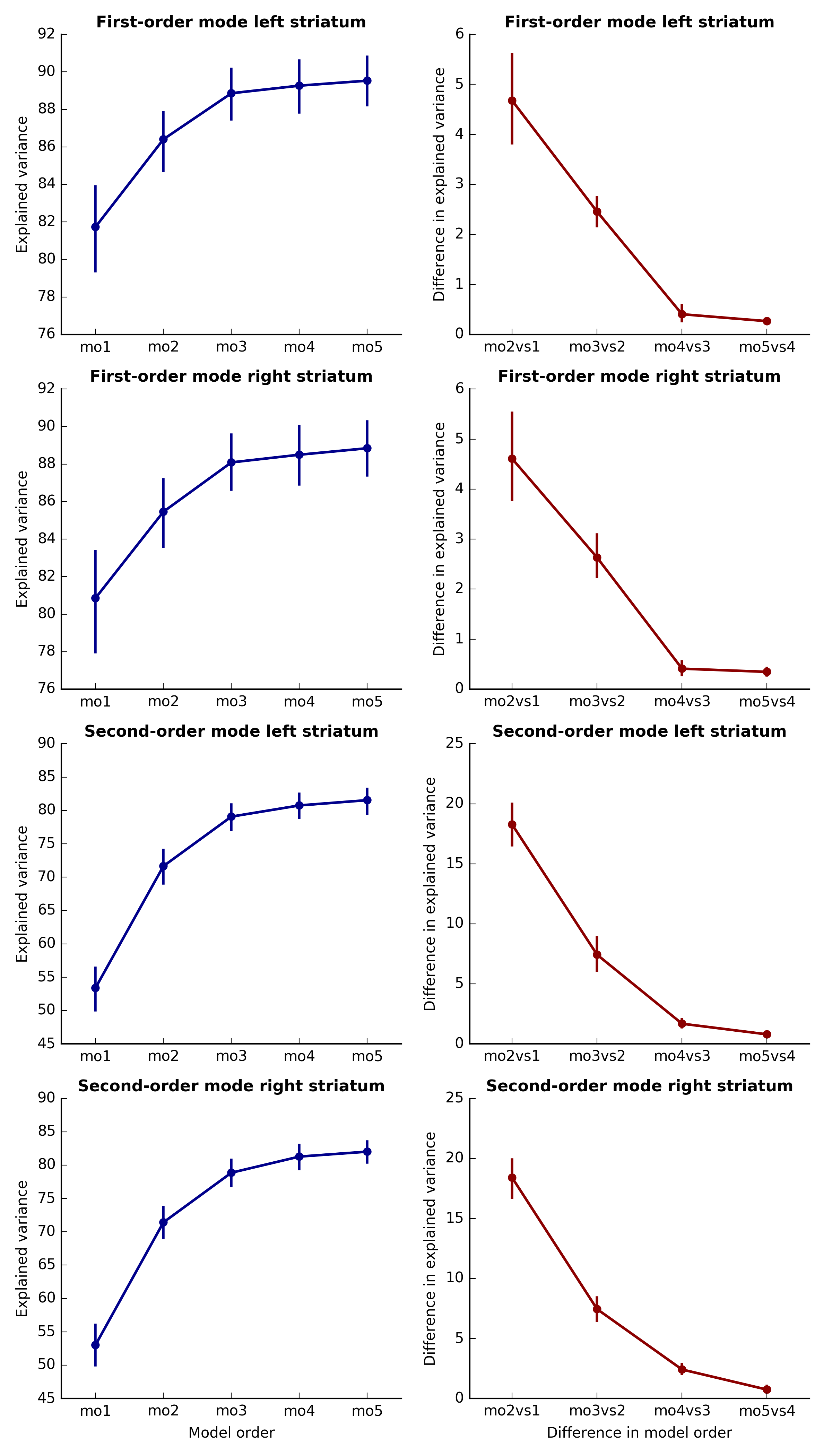


# Figure S3. The variance explained by TSM models ranging from model order 1 to 5 applied to the four connectivity modes in the FEP dataset. **Abbreviations: mo = model order, vs = versus.**

#
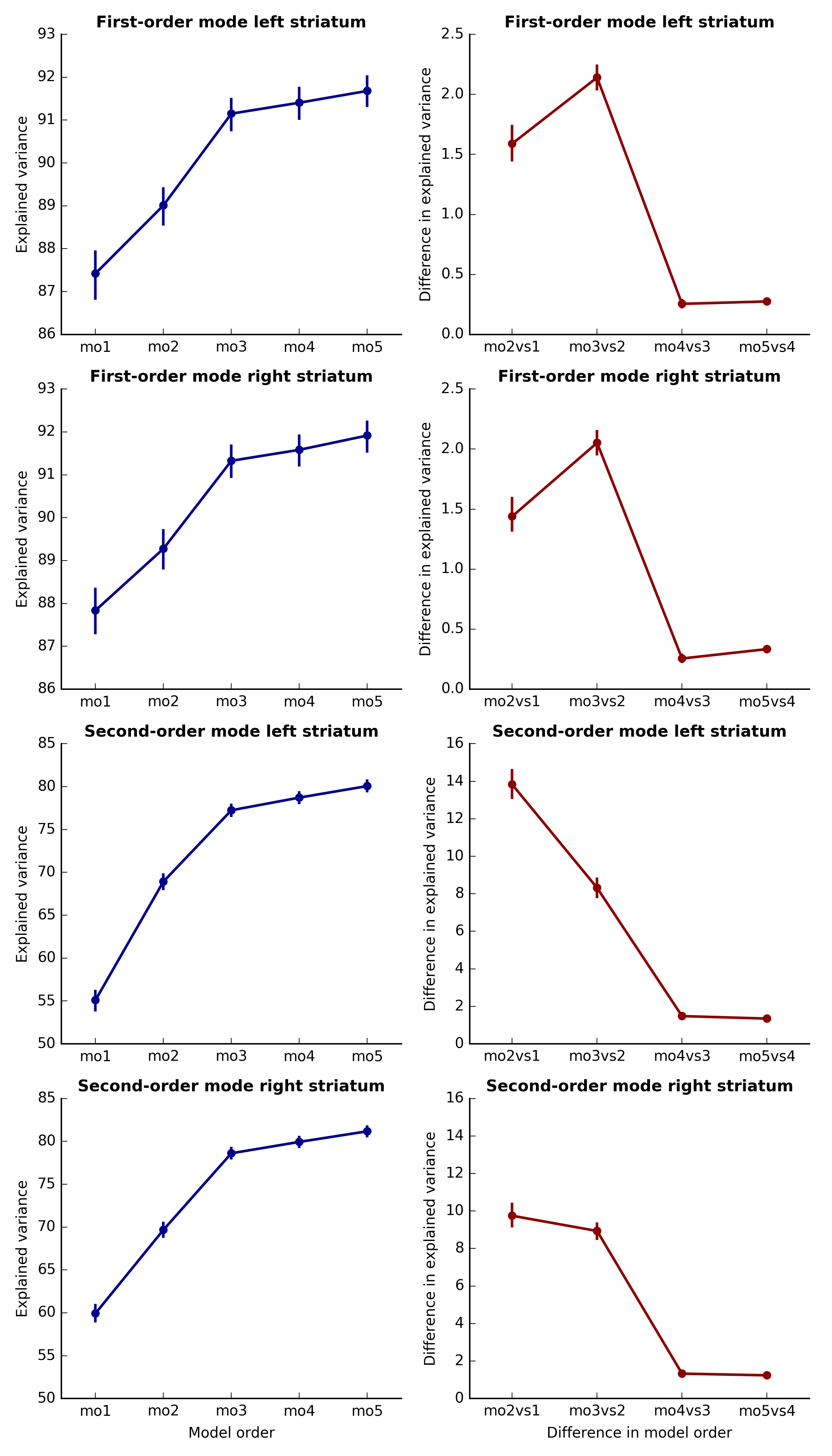


# Figure S4. The variance explained by TSM models ranging from model order 1 to 5 applied to the four connectivity modes in the community dataset. **Abbreviations: mo = model order, vs = versus.**

# Supplementary References

1. Pinar A, Hawi Z, Cummins T, Johnson B, Pauper M, Tong J, et al. Genome-wide association study reveals novel genetic locus associated with intra-individual variability in response time. 2018;8:1-8.

2. Qualtrics: Qualtrics. Provo, Utah, USA2005.

3. Peters E, Joseph S, Day S, Garety P. Measuring delusional ideation: the 21-item Peters et al. Delusions Inventory (PDI). Schizophrenia bulletin. 2004;30:1005-1022.

4. Eckblad M, Chapman LJ. Magical ideation as an indicator of schizotypy. Journal of consulting and clinical psychology. 1983;51:215.

5. Chapman LJ, Chapman JP, Raulin ML. Scales for physical and social anhedonia. Journal of abnormal psychology. 1976;85:374.

6. Chapman LJ, Chapman JP, Raulin ML. Body-image aberration in schizophrenia. Journal of abnormal psychology. 1978;87:399.

7. Stefanis N, Hanssen M, Smirnis N, Avramopoulos D, Evdokimidis I, Stefanis C, et al. Evidence that three dimensions of psychosis have a distribution in the general population. Psychological medicine. 2002;32:347-358.

8. Mason O, Linney Y, Claridge G. Short scales for measuring schizotypy. Schizophrenia research. 2005;78:293-296.

9. Bollen KA, Noble MD. Structural equation models and the quantification of behavior. Proceedings of the National Academy of Sciences. 2011;108:15639-15646.

10. IBM Corp: IBM SPSS Statistics for Windows. 27.0 ed. Armonk, NY, IBM Corp; Released 2020.

11. Enders CK: Applied missing data analysis. Little TD, editor. New York, The Guilford Press; 2010.

12. Muthén LK, Muthén BO: Mplus User’s Guide. Eighth ed. Los Angeles, CA, Muthén & Muthén; 1998 - 2017.

13. Kline RB: Principles and practice of structural equation modeling. 4th ed. Kenny DA, Little TD, editors. New York, The Guilford Press; 2015.

14. Silvia ESM, MacCallum RC. Some factors affecting the success of specification searches in covariance structure modeling. Multivariate Behavioral Research. 1988;23:297 - 326.

15. Benjamini Y, Hochberg Y. Controlling the false discovery rate: A practical and powerful approach to multiple testing. Journal of the Royal Statistical Society Series B (Methodological). 1995;57:289-300.

16. Marsh HW, Hau KT, Wen ZL. In search of golden rules: Comment on hypothesis-testing approaches to setting cutoff values for fit indexes and dangers in overgeneralizing Hu and Bentler's (1999) findings. Struct Equ Modeling. 2004;11:320-341.

17. Hayduk L, Cummings G, Boadu K, Pazderka-Robinson H, Boulianne S. Testing! testing! one, two, three - Testing the theory in structural equation models! Pers Indiv Differ. 2007;42:841-850.

18. Hair JF, Black WC, Babin BJ, Anderson RE: Multivariate data analysis. Seventh edition, Pearson new international edition. ed2014.

19. Byrne BM, Shavelson RJ, Muthen B. Testing for the Equivalence of Factor Covariance and Mean Structures - the Issue of Partial Measurement Invariance. Psychol Bull. 1989;105:456-466.

20. Barrett PQ. Structural equation modelling: Adjudging model fit. Pers Indiv Differ. 2007;42:815-824.

21. Kline RB: Principles and practice of structural equation modeling. 4th edition. ed2015.

22. Hair JF, Black WC, Babin BJ, Anderson RE: Multivariate data analysis. Seventh ed. Edinburgh Gate, Pearson Education Limited; 2014.

23. Wagenmakers EJ. A practical solution to the pervasive problems of p values. Psychon B Rev. 2007;14:779-804.

24. Kass RE, Raftery AE. Bayes factors. Journal of the American Statistical Association. 1995;90:773-795.

25. Raftery AE. Bayesian model selection in social research. Sociol Methodol. 1995;25:111-163.

26. Stark S, Chernyshenko OS, Drasgow F. Detecting differential item functioning with confirmatory factor analysis and item response theory: Toward a unified strategy. Journal of Applied Psychology. 2006;91:1292 - 1306.

27. Byrne BM: Structural equation modeling with Mplus: Basic concepts, applications, and programming. New York, Routledge; 2012.

28. Meredith W. Measurement Invariance, Factor-Analysis and Factorial Invariance. Psychometrika. 1993;58:525-543.

29. Putnick DL, Bornstein MH. Measurement invariance conventions and reporting: The state of the art and future directions for psychological research. Developmental Review. 2016;41:71-90.

30. Yoon M, Kim ES. A comparison of sequential and nonsequential specification searches in testing factorial invariance. Behavior Research Methods. 2014;46:1199-1206.

31. Grice JW. Computing and evaluating factor scores. Psychological methods. 2001;6:430.

32. Peters ER, Joseph S, Day S, Garety P. Measuring delusional ideation: The 21-item Peters et al. Delusions Inventory (PDI). Schizophrenia Bulletin. 2004;30:1005 - 1022.

33. Mason O, Linney Y, Claridge G. Short scales for measuring schizotypy. Schizophrenia Research. 2005;78:293 - 296.

34. Kwapil TR, Barrantes-Vidal N, Silvia PJ. The dimensional structure of the Wisconsin schizotypy scales: Factor identification and construct validity. Schizophrenia Bulletin. 2007;34:444-457.

35. Brenner K, Schmitz N, Pawliuk N, Fathalli F, Joober R, Ciampi A, et al. Validation of the English and French versions of the Community Assessment of Psychic Experiences (CAPE) with a Montreal community sample. Schizophr Res. 2007;95:86-95.

36. Oldehinkel M, Llera A, Faber M, Huertas I, Buitelaar JK, Bloem BR, et al. Mapping dopaminergic projections in the human brain with resting-state fMRI. eLife. 2022;11:e71846.

37. Marquand AF, Haak KV, Beckmann CF. Functional corticostriatal connection topographies predict goal-directed behaviour in humans. Nature human behaviour. 2017;1:s41562-41017-40146.
